# Expression of *mecA* increases daptomycin tolerance in *Staphylococcus aureus*

**DOI:** 10.1128/mbio.02250-25

**Published:** 2025-09-22

**Authors:** Elizabeth V. K. Ledger, Mario Recker, Ruth C. Massey

**Affiliations:** 1 School of Microbiology, University College Cork 8795, Cork, Ireland; 2 APC Microbiome Ireland, University College Cork 8795, Cork, Ireland; 3 Centre for Ecology and Conservation, University of Exeter, Penryn Campus 151778, Penryn, United Kingdom; 4 Institute for Tropical Medicine, University of Tübingen 9188, Tübingen, Germany; 5 School of Cellular and Molecular Medicine, University of Bristol 1980, Bristol, United Kingdom; University of Pretoria, Pretoria, Gauteng, South Africa

**Keywords:** *Staphylococcus aureus*, daptomycin, *mecA*, PBP2a, antibiotic tolerance, toxins

## Abstract

*Staphylococcus aureus* is a leading cause of bacteremia, and infections caused by methicillin-resistant *S. aureus* (MRSA) strains are especially challenging to treat. MRSA strains are resistant to front-line beta-lactams due to PBP2a, a low-affinity penicillin-binding protein encoded by *mecA*. Daptomycin is used to treat MRSA infections but is not always effective. While daptomycin resistance has been well studied, the ability of *S. aureus* to tolerate daptomycin, a feature likely to slow clearance of the bacteria from the bloodstream, is less well understood. Here, using a panel of clinical bacteremia isolates, we show that MRSA strains are more tolerant of daptomycin than methicillin-susceptible *S. aureus* (MSSA) strains. This difference in tolerance is due to *mecA* and is independent of any changes in surface properties previously associated with altered daptomycin susceptibility. Instead, using a *mecA* transposon mutant, we found that a lack of this gene led to higher activity of the Agr quorum sensing system, resulting in an increased release of the phenol-soluble modulin toxins. Increased levels of these surfactant-like toxins prevented daptomycin from being inactivated by lipids released by the bacteria, leading to reduced antibiotic tolerance. Additionally, the clinical MRSA strains tested produced lower levels of toxins than the MSSA strains and inactivated daptomycin to a greater extent, explaining their enhanced tolerance. The expression of *mecA* in clinical MSSA strains reduced toxin production, increasing daptomycin inactivation and thereby enhancing tolerance. Together, these results demonstrate that *mecA* not only affects beta-lactam susceptibility but also compromises the efficacy of the last resort antibiotic daptomycin.

**IMPORTANCE:**

The incidence of *Staphylococcus aureus* bacteremia is on a steady incline in many parts of the world. Given the associated mortality rates have changed little in the last 10 years, this is a major health concern. One contributing problem is that antibiotics effective against the bacteria *in vitro* are failing to cure many patients, e.g., the use of daptomycin to treat methicillin-resistant *Staphylococcus aureus* (MRSA) infections. Here, we present a mechanistic study that may explain why this occurs. The expression of *mecA*, which confers the methicillin resistance of MRSA, reduces Agr activity, and this decreases the release of phenol-soluble modulins from the bacteria. Without these, the phospholipids that can block daptomycin activity are free to do so, rendering MRSA less sensitive to both antibiotics. This study, bridging clinical and molecular biology, provides an explanation for a significant clinical problem and may inform how antibiotic combinations should be managed in future trials.

## INTRODUCTION

*Staphylococcus aureus* is a major human pathogen, causing a range of infections from minor skin and soft tissue infections to life-threatening diseases like bacteremia and endocarditis (1). It is the most common causative agent of skin infections and is the leading Gram-positive cause of bacteremia, responsible for over 14,000 cases in 2023 in the UK (2). *S. aureus* bacteremia requires urgent intravenous antibiotics to sterilize the bloodstream, has a mortality rate of approximately 25% (3), and can lead to the development of secondary infections, including deep tissue abscesses and endocarditis (4).

The treatment prescribed depends on the antibiotic susceptibility of the strain causing the infection, with methicillin-susceptible *S. aureus* (MSSA) treated with front-line beta-lactams such as flucloxacillin or cefazolin (5, 6). Treatment options for methicillin-resistant *S. aureus* (MRSA) are more limited but include vancomycin and daptomycin (5, 6). Regardless of the susceptibility of the pathogen, long-term IV antibiotics are needed, with a minimum of a 6-week course recommended in cases of complicated bacteremia (7). Importantly, the time to the administration of appropriate antibiotics is also critical, and delays to this can lead to increases in 30-day mortality rates (8).

Vancomycin can be challenging to dose correctly as it can require labor-intensive monitoring to ensure the correct serum concentrations are achieved (9). These limitations mean that daptomycin is preferred in some cases. Daptomycin is a lipopeptide antibiotic that binds to membrane phosphatidylglycerol in a calcium-dependent manner (10, 11). This disrupts membrane integrity and cell wall synthesis, leading to cell death (12–14). Daptomycin is rapidly bactericidal *in vitro,* and resistance is extremely rare, with over 99.9% of infections caused by daptomycin-susceptible strains (15). However, despite this, daptomycin suffers from high rates of treatment failure (16, 17). Understanding the reasons behind this is crucial to improving patient outcomes.

A range of approaches has been used to identify factors that affect daptomycin resistance, including studying paired clinical isolates from before and after daptomycin therapy (18–20) and generating resistant mutants *in vitro* (21). Together, these have found that daptomycin susceptibility can be influenced by changes in the cell membrane, such as an increased positive charge resulting from increased lysylphosphatidylglycerol content or altered fatty acid compositions resulting in changes to membrane fluidity (22–25). Increases in cell wall thickness and wall teichoic acid (WTA) content have also been found to reduce daptomycin susceptibility (18, 26). Daptomycin tolerance, which is distinct from resistance and is defined as a slower rate of bacterial killing, is also thought to contribute to treatment failure as it delays the clearance of the bacteria from the bloodstream (27). This tolerance can be phenotypic and induced by the host environment (28, 29), or due to mutations in genes such as those encoding the PitA phosphate transporter (30) or the alkaline shock protein Asp23 (31). *S. aureus* can also tolerate exposure to daptomycin through the release of membrane phospholipids that bind to daptomycin, sequestering it and preventing it from killing the bacteria (32). However, this interaction between lipids and daptomycin is compromised by the release of phenol-soluble modulins (PSMs), small surfactant-like toxins whose production is controlled by the accessory gene regulator (Agr) quorum sensing system (32).

While much is known about the factors affecting daptomycin susceptibility that arises in both clinical and laboratory-based environments, most of what is known about daptomycin tolerance is laboratory based, and the exact mechanisms used by *S. aureus* to tolerate daptomycin in clinical situations remain to be determined. As such, here we focused on collections of clinical isolates, with the aim of discovering genes that contribute to tolerance in clinically relevant situations, with the longer-term goal of identifying therapeutic targets to improve daptomycin treatment efficacy.

## RESULTS

### MRSA strains are more tolerant of daptomycin than MSSA strains

As a first step to identifying factors that affect the tolerance of *S. aureus* to daptomycin, we studied a collection of 300 *S. aureus* bacteremia clinical isolates belonging to either clonal complex (CC) 22 or 30 (CC22, *n*  =  135; CC30, *n*  =  165), which contained both MRSA and MSSA isolates (33). These strains were isolated from a single hospital in the UK between 2006 and 2012. An overnight culture of each strain was adjusted to 1 × 10^8^ CFU mL^−1^ and exposed to 10 µg mL^−1^ daptomycin (corresponding to mean serum levels achieved in patients (34) for 2 h and survival measured by colony forming unit (CFU) counts.

**Fig 1:**
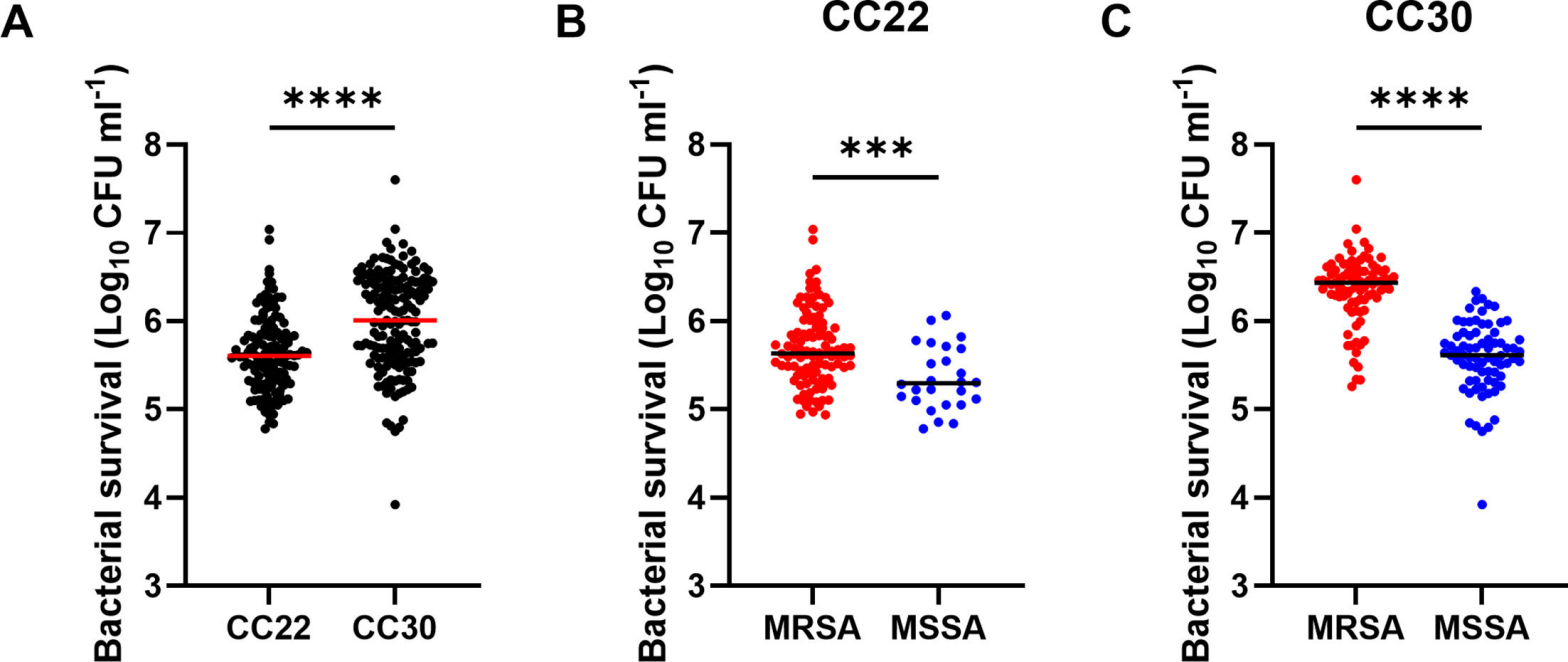
MRSA strains are more tolerant of daptomycin than MSSA strains. (**A**) Clinical isolates of *S. aureus* from CC22 (*n* = 135) or from CC30 (*n* = 165) at 10^8^ CFU mL^−1^ were exposed to 10 µg mL^−1^ daptomycin for 2 h before survival was determined by CFU counts. (**B–C**) Data from A are re-plotted with strains from (**B**) CC22 and (**C**) CC30 separated based on susceptibility to methicillin. Each data point represents the mean survival of three independent repeats of one strain, and the median of all the strains is indicated. Data were analyzed by an unpaired *t*-test (***, *P* ≤ 0.001. ****, *P* ≤ 0.0001).

Daptomycin resistance is rare (15), and so, as expected, the CFU counts of each of the strains decreased on exposure to the antibiotic (Fig. 1A). However, the survival of the strains varied, with a >3 log difference in survival between the most and least susceptible isolates. There were differences in survival between the clonal complexes, with the median survival of the isolates from CC22 and CC30 being 4 × 10^5^ and 1 × 10^6^ CFU mL^−1^, respectively (Fig. 1A). Additionally, there was a significant difference in survival between MRSA and MSSA strains, where the MRSA isolates survived exposure to daptomycin better than the MSSA isolates (2.2- and 5.8-fold in CC22 and CC30, respectively) (Fig. 1B and C). As a first step to investigate this difference in antibiotic susceptibility, we measured the daptomycin minimum inhibitory concentration (MIC) of each of these 300 clinical isolates (Table S1). This revealed that the vast majority of strains (95.7%) had a MIC of 0.5 µg mL^−1^. There was no difference in MIC observed between the clonal complexes, with 94.5% of CC30 strains and 97% of CC22 strains having a MIC of 0.5 µg mL^−1^. Additionally, differences in MIC did not explain the difference in daptomycin tolerance between MRSA and MSSA isolates. Together, this suggests that the CC30 strains are more daptomycin tolerant than the CC22 strains, and MRSA strains from both clonal complexes show increased daptomycin tolerance compared to MSSA strains.

### Expression of *mecA* increases tolerance of JE2 and clinical MSSA isolates to daptomycin

**Fig 2:**
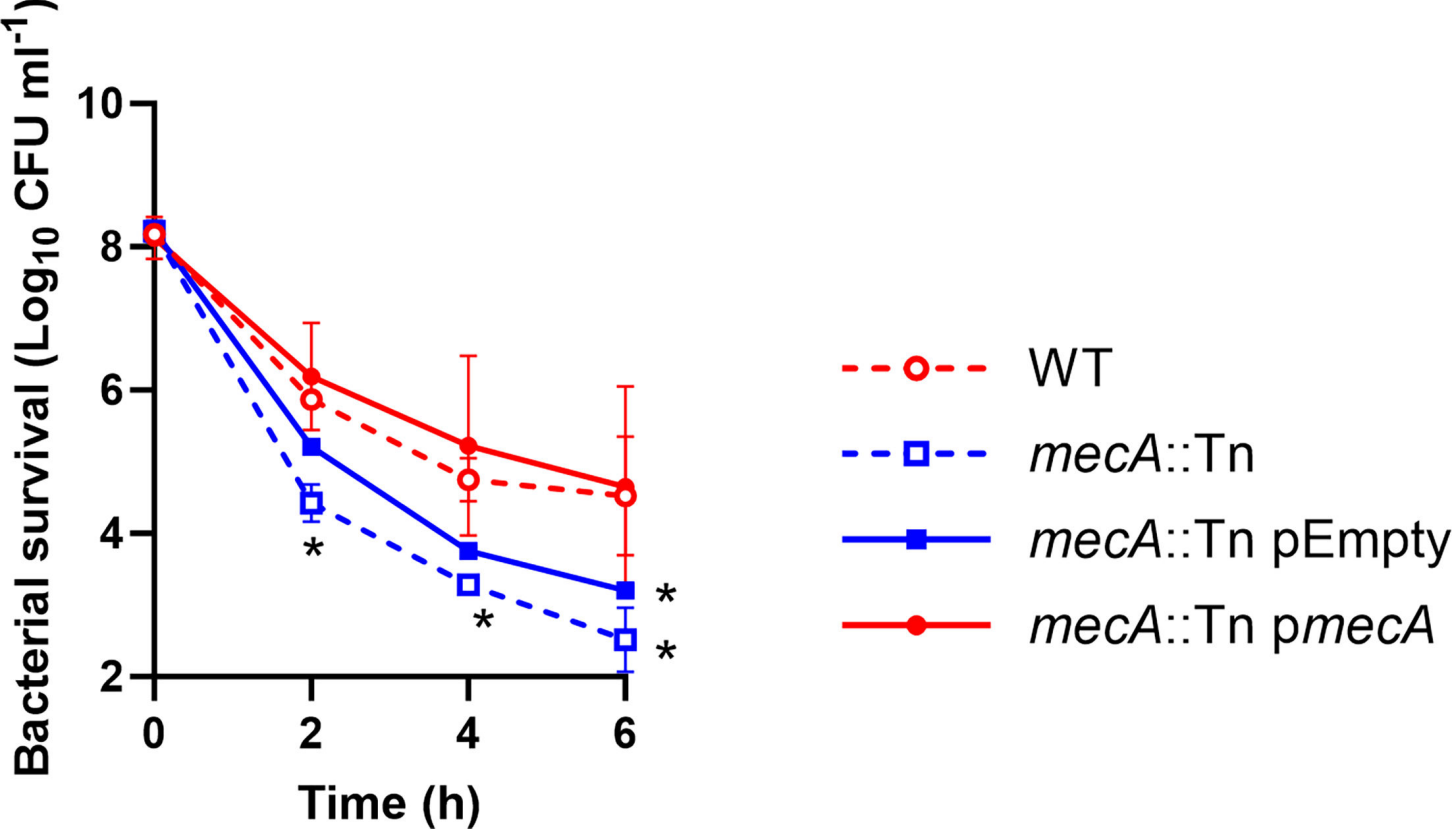
Expression of *mecA* increases the tolerance of JE2 to daptomycin. Survival of JE2 WT, *mecA*::Tn, *mecA*::Tn complemented with an empty plasmid, or *mecA*::Tn complemented with p*mecA* during a 6 h exposure to 10 µg mL^−1^ daptomycin (Log_10_ CFU mL^−1^). Data represent the mean ± standard deviation of three independent biological repeats. Data were analyzed by two-way ANOVA with Sidak’s post-hoc test. *, *P* < 0.05 (WT vs mutants at indicated time points).

Daptomycin and beta-lactam resistance are typically negatively correlated, with resistance to daptomycin sensitizing strains to beta-lactams, a phenomenon known as the “seesaw effect” (35, 36). Therefore, we aimed to understand why this negative correlation was not observed here, and why MRSA strains were more tolerant of daptomycin than MSSA strains. First, we investigated whether the difference in daptomycin tolerance between MRSA and MSSA strains was due to the presence of the *mecA* gene in MRSA strains, which encodes the low-affinity penicillin-binding protein (PBP), PBP2a. To do this, we exposed *S. aureus* JE2 wildtype (WT) and the *mecA*::Tn mutant from the Nebraska Transposon mutant library (NTML) (37) to daptomycin for 6 h and measured survival over time by CFU counts. This demonstrated that the *mecA*::Tn mutant was approximately 100-fold less tolerant to the antibiotic than the WT strain (Fig. 2). To confirm that this was due to *mecA* and not polar effects of the transposon insertion, we complemented the *mecA*::Tn mutant with a WT copy of *mecA* under the control of the tetracycline-inducible promoter on p*itet,* a plasmid which integrates into the genome at the *geh* locus (38). Complementation of the *mecA*::Tn mutant with this plasmid (p*mecA*) restored daptomycin tolerance to WT levels, while complementation with the empty vector control did not (Fig. 2). Therefore, loss of *mecA* decreases the tolerance of *S. aureus* to daptomycin.

**Fig 3:**
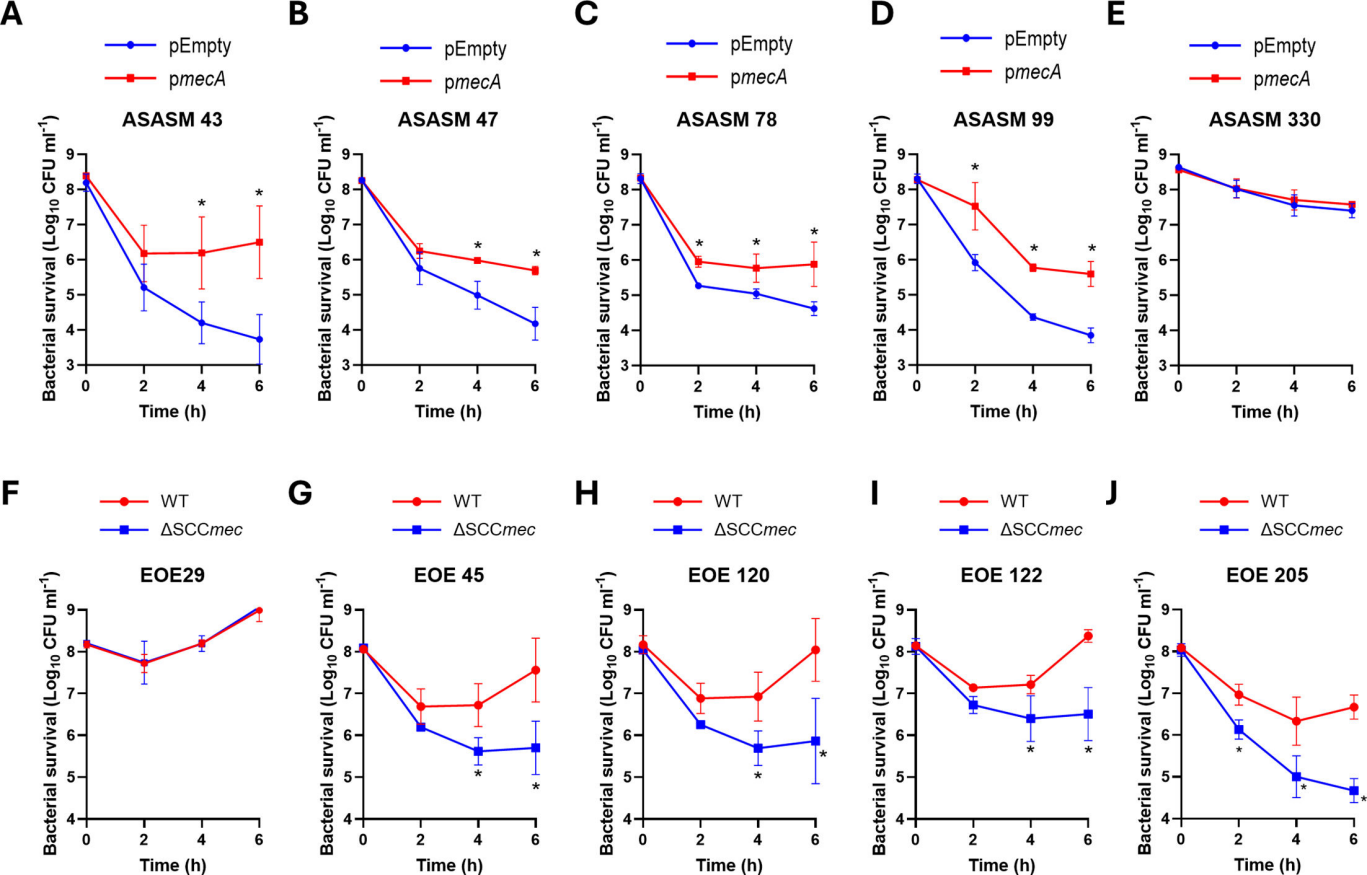
Expression of *mecA* affects the tolerance of clinical isolates to daptomycin. Survival of (**A**) ASASM 43, (**B**) ASASM 47, (**C**) ASASM 78, (**D**) ASASM 99, and (**E**) ASASM 330 complemented with either the empty plasmid (blue) or p*mecA* (red) during a 6 h exposure to 10 µg mL^−1^ daptomycin. Survival of (**F**) EOE 29, (**G**) EOE 45, (**H**) EOE 120, (**I**) EOE 122, and (**J**) EOE 205 WT (red) and with the SCC*mec* element deleted (ΔSCC*mec*; blue) during a 6 h exposure to 10 µg mL^−1^ daptomycin. Data represent the mean ± standard deviation of three independent biological repeats. Data were analyzed by two-way ANOVA with Sidak’s post-hoc test. *, *P* < 0.05 (pEmpty vs p*mecA* [**A–E**] or WT vs ΔSCC*mec* [**F–J**] at indicated time points).

Next, we aimed to investigate whether expression of *mecA* also affected daptomycin tolerance in clinical isolates. As a larger difference in daptomycin tolerance was observed between MRSA and MSSA strains from CC30 than CC22, we chose to characterize the mechanism behind this phenotype using strains from this clonal complex. First, we transformed the empty plasmid or p*mecA* into five representative MSSA strains from CC30, which spanned the observed range of daptomycin susceptibilities (the strains chosen are indicated in Fig. S1). Introduction of *pmecA* into these strains and induction with anhydrotetracycline did not affect their cloxacillin or cefoxitin MICs, likely as additional mutations are needed to confer beta-lactam resistance (39, 40), and did not affect the daptomycin MIC (Table S2). However, daptomycin was rapidly bactericidal against four out of five of these strains containing the empty plasmid, causing between 3 and 4 logs of killing by 6 h (Fig. 3A through D). In each of these strains, induction of *mecA* expression led to a significant reduction in antibiotic killing (Fig. 3A through D). The fifth strain tested, ASASM330, was more tolerant than the others, with only 1 log of killing observed by 6 h and, in this case, induction of *mecA* expression had no effect on daptomycin tolerance (Fig. 3E). We also confirmed that induction of *mecA* expression increased daptomycin tolerance in an additional MSSA strain, ATCC 29213, a strain commonly used in antimicrobial susceptibility testing (Fig. S2). Expression of *mecA* in this strain led to a >4 log increase in bacterial survival on daptomycin exposure at 6 h compared to a strain containing the empty plasmid (Fig. S2).

Additionally, we deleted the SCC*mec* element (containing *mecA*) from five representative clinical MRSA strains, which spanned the observed range of daptomycin susceptibilities (indicated in Fig. S1) and investigated the effect of this on daptomycin susceptibility. In each case, except for one of the strains (EOE 29), deletion of the SCC*mec* element sensitized strains to daptomycin, with a 1–2 log increase in killing observed by 6 h (Fig. 3F through J). Taken together, *mecA* affects daptomycin susceptibility in both the laboratory strain JE2 and clinical isolates, with a lack of *mecA* expression decreasing tolerance, and increased *mecA* expression resulting in increased tolerance.

### PBP2a does not affect daptomycin susceptibility by altering surface properties

The next objective was to understand how the expression of *mecA* affected daptomycin tolerance. Many factors have been identified to impact daptomycin susceptibility/resistance, including increased cell-wall thickness, alterations to the cell membrane, and increased positive charge on the cell surface (11). Therefore, we next tested whether any of these properties differed between five representative MRSA and MSSA strains and whether these differences depended on *mecA*.

**Fig 4:**
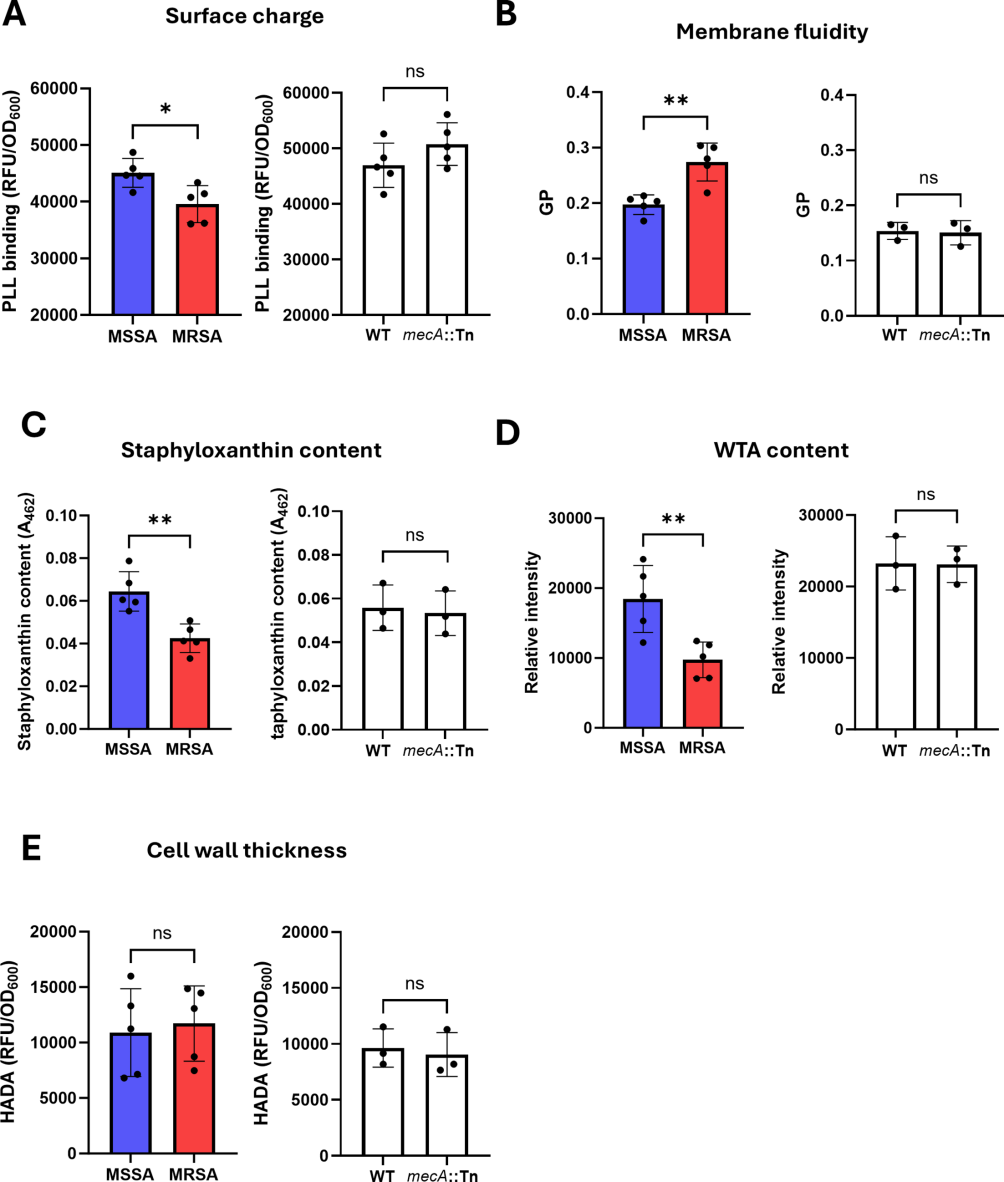
PBP2a does not affect daptomycin tolerance by altering surface properties. (**A**) Surface charge of five MSSA and five MRSA strains (left) and JE2 WT and the *mecA*::Tn mutant (right) as determined by binding of FITC-labeled PLL. Fluorescence values were divided by OD_600_ to normalize for difference in cell density. (**B**) Membrane fluidity of five MSSA and five MRSA strains (left) and JE2 WT and the *mecA*::Tn mutant (right) as determined by the generalized polarization index obtained by measuring fluorescence of laurdan. (**C**) Staphyloxanthin content of five MSSA and five MRSA strains (left) and JE2 WT and the *mecA*::Tn mutant (right). (**D**) Wall teichoic acid content of five MSSA and five MRSA strains (left) and JE2 WT and the *mecA*::Tn mutant (right) as determined by WTA extraction, analysis by native PAGE and visualization with alcian blue. Intensity was quantified using ImageJ. (**E**) Cell-wall thickness of five MSSA and five MRSA strains (left) and JE2 WT and the *mecA*::Tn mutant (right) as determined by the incorporation of the fluorescent D-amino acid analog HADA. Fluorescence values were divided by OD_600_ to normalize for difference in cell density. In the left-hand graph of each, each data point represents the mean of three independent repeats of one strain. In the right-hand graph of each, data represent the mean ± standard deviation of at least three independent repeats. Data were analyzed by *t*-test. *, *P* ≤ 0.05. **, *P* ≤ 0.01.

First, we examined surface charge, using a fluorescently labeled positively charged molecule, poly-L-lysine (FITC-PLL). This demonstrated that the MRSA strains were more positively charged than the MSSA strains tested and so would be expected to show increased repulsion of the positively charged antibiotic (Fig. 4A). However, the difference in surface charge was not dependent on PBP2a*,* with a *mecA*::Tn mutant having the same surface charge as the WT strain (Fig. 4A). Similarly, we observed differences in membrane fluidity, staphyloxanthin content, and WTA levels between the MRSA and MSSA strains, but none of these differences were dependent on *mecA* (Fig. 4B through D). There were no differences observed in cell-wall thickness between the MSSA and MRSA strains or between WT and the *mecA*::Tn mutant (Fig. 4E). Therefore, the *mecA*-dependent increased daptomycin tolerance of MRSA strains compared with MSSA could not be explained by differences in cell surface charge, membrane fluidity, staphyloxanthin levels, WTA content, or cell-wall thickness.

### Expression of *mecA* increases daptomycin tolerance by increasing inactivation of the antibiotic

Tolerance to daptomycin has previously been linked to activity of the Agr quorum sensing system, with Δ*agr* mutants showing reduced susceptibility compared to the WT strain (32). This is because *S. aureus* releases phospholipids, which sequester daptomycin, inactivating it. This binding of phospholipids to daptomycin is compromised by the production of PSMs, small surfactant-like toxins whose expression is controlled by Agr (32). To understand how *mecA* affects daptomycin susceptibility, we first confirmed that an *agrA*::Tn mutant showed increased daptomycin tolerance and increased antibiotic inactivation under our conditions (Fig. S3). Next, we determined whether this could explain the observed difference in daptomycin susceptibility between MRSA and MSSA strains.

**Fig 5:**
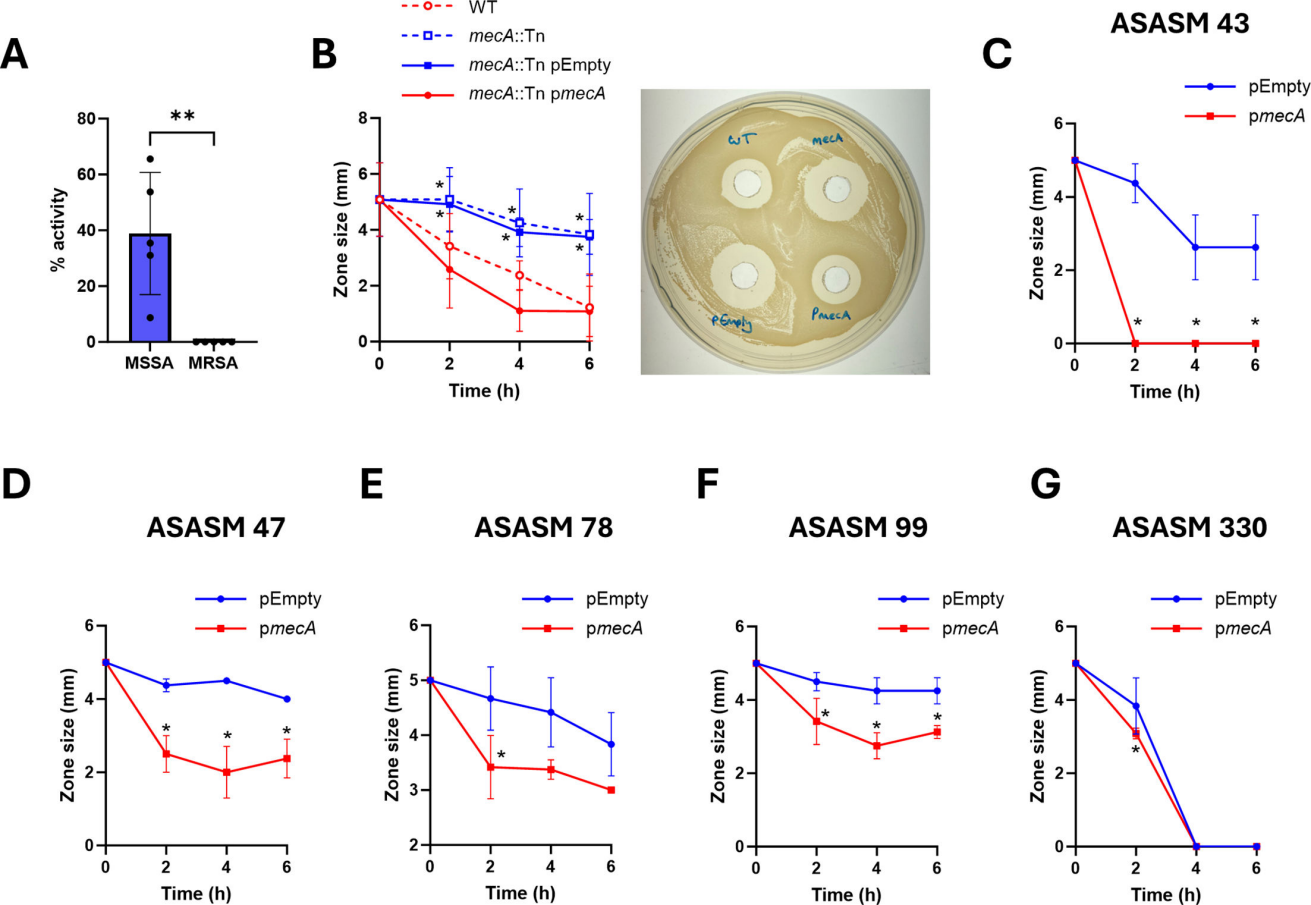
Expression of *mecA* increases daptomycin tolerance by increasing inactivation of the antibiotic. (**A**) Percentage of daptomycin activity remaining in the supernatant as measured by a zone of inhibition assay after exposure of five MSSA strains and five MRSA strains to 10 µg mL^−1^daptomycin for 6 h. (**B**) Left: Daptomycin activity remaining in the supernatant after exposure of JE2 WT, *mecA*::Tn, *mecA*::Tn complemented with an empty plasmid or *mecA*::Tn complemented with p*mecA* to 10 µg mL^−1^ daptomycin. Right: Representative image of zones of inhibition at the 6 h time point. Daptomycin activity remaining in the supernatant after exposure of (**C**) ASASM 43, (**D**) ASASM 47, (**E**) ASASM 78, (**F**) ASASM 99, and (**G**) ASASM 330 complemented with either the empty plasmid (blue) or p*mecA* (red) to 10 µg mL^−1^ daptomycin. In A, each data point represents the mean of three independent repeats of one strain, and data were analyzed by *t*-test. All other graphs represent the mean ± standard deviation of three independent repeats. Data were analyzed by two-way ANOVA with Sidak’s *post-hoc* test. *, *P* ≤ 0.05, **, *P* ≤ 0.01 (MRSA vs MSSA in A; WT vs other strains at indicated time-points in B; pEmpty vs p*mecA* at indicated time points in C–G).

We measured whether there were differences in the degree to which daptomycin was inactivated by the MRSA strains versus the MSSA strains. To do this, the strains were exposed to daptomycin for 6 h before the activity of daptomycin remaining in the supernatant was quantified using a zone of inhibition assay. This revealed that all five of the MRSA strains fully inactivated daptomycin, whereas the MSSA strains inactivated daptomycin to a lesser degree, with an average of 40% of the starting activity of daptomycin remaining in the supernatant after 6 h (Fig. 5A). The ability of *S. aureus* to inactivate daptomycin was dependent on *mecA,* with a *mecA*::Tn mutant inactivating significantly less daptomycin over 6 h than the WT strain (Fig. 5B). Complementation of the *mecA*::Tn mutant with a plasmid expressing *mecA*, but not with an empty plasmid control, restored daptomycin inactivation to WT levels (Fig. 5B).

Next, we investigated whether induction of *mecA* expression in the MSSA strains affected their ability to inactivate daptomycin. In line with the daptomycin tolerance data in Fig. 3A through E, expression of *mecA* in each of the MSSA strains, except ASASM330, led to increased daptomycin inactivation compared with the empty plasmid control (Fig. 5C through G). ASASM330 fully inactivated daptomycin in the absence of *mecA*, explaining the high tolerance to daptomycin observed in Fig. 3E (Fig. 5G). Taken together, the expression of *mecA* increases the ability of *S. aureus* to inactivate daptomycin, resulting in reduced susceptibility to the antibiotic.

### Expression of *mecA* increases daptomycin inactivation by reducing production of PSMs.

**Fig 6:**
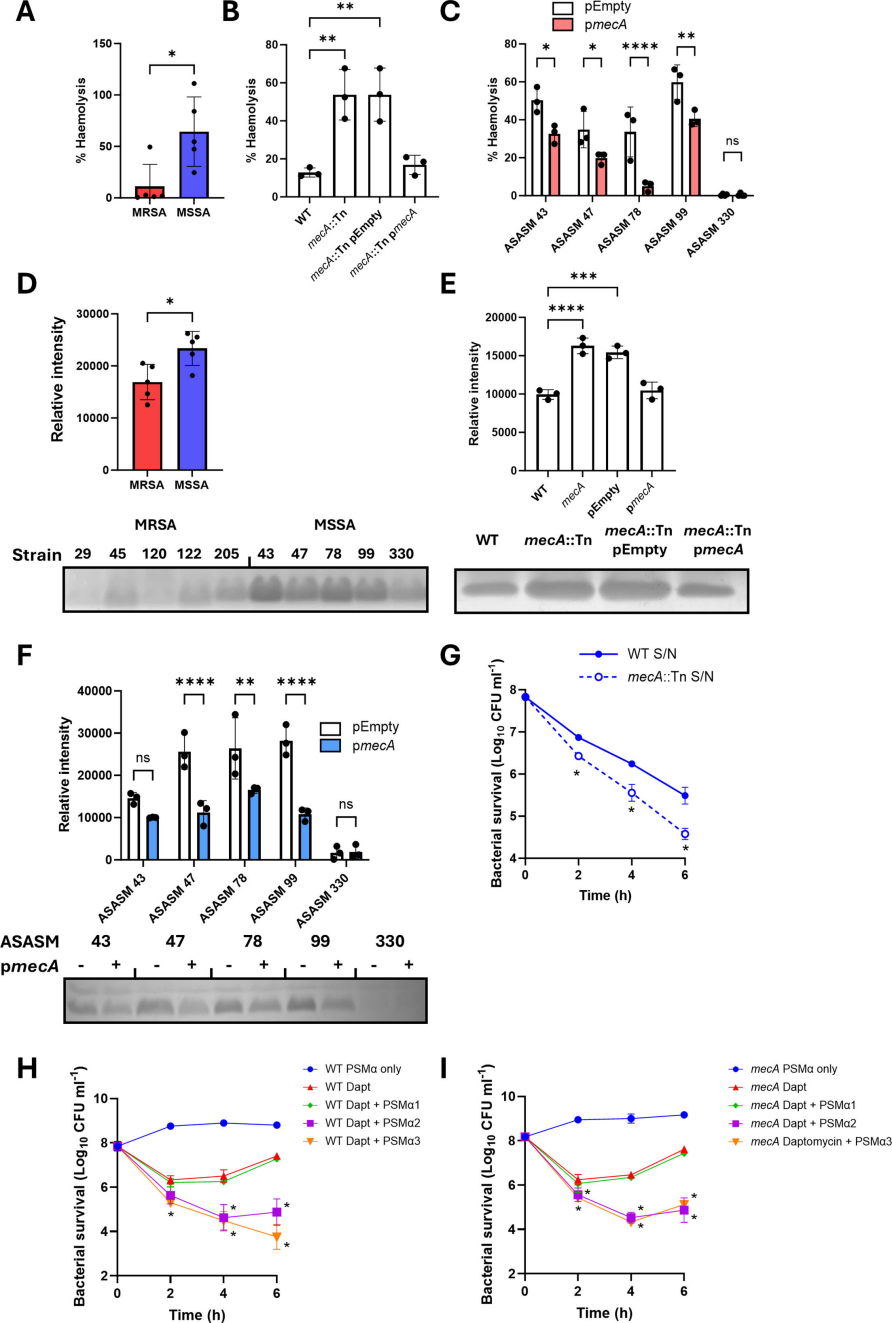
Expression of *mecA* increases daptomycin inactivation by reducing production of PSMs. (**A**) Percent hemolysis of sheep erythrocytes by supernatants from five MRSA and five MSSA strains. (**B**) Percent hemolysis caused by supernatants from JE2 WT, *mecA*::Tn and *mecA*::Tn complemented with the empty plasmid or with p*mecA*. (**C**) Percent hemolysis of supernatants from five MSSA strains complemented with either the empty plasmid or with p*mecA*. (**D**) Supernatants from five MRSA and five MSSA strains were analyzed by SDS-PAGE to visualize and quantify the PSMs. (**E**) Supernatants from JE2 WT, *mecA*::Tn and *mecA*::Tn complemented with the empty plasmid or with p*mecA* were analyzed by SDS-PAGE to visualize and quantify the PSMs. (**F**) Supernatants from five MSSA strains complemented with either the empty plasmid or with p*mecA* were analyzed by SDS-PAGE to visualize and quantify the PSMs. (**G**) Log_10_ CFU mL^−1^ of JE2 WT during a 6-h exposure to 10 µg mL^−1^ daptomycin in spent culture supernatant from either the WT strain or from the *mecA*::Tn mutant. Survival of (**H**) JE2 WT and (**I**) the *mecA*::Tn mutant during a 6-h exposure to 10 µg mL^−1^ daptomycin in media supplemented, or not, with 10 µM purified PSMs. In A and D, each data point represents the mean of three independent repeats of one strain and data were analyzed by *t*-test. Data in B, C, E, F, G, H, and I represent the mean ± standard deviation of three independent repeats. Data in B and E were analyzed by one-way ANOVA with Dunnett’s post-hoc test. Data in C, F, G, H, and I were analyzed by two-way ANOVA with Sidak’s *post-hoc* test. *, *P* ≤ 0.05; **P ≤ 0.01; ***P ≤ 0.001; ****P ≤ 0.0001. In D, E, and F, representative images are shown from three repeats.

The final objective was to determine how expression of *mecA* affected daptomycin inactivation. Previous work has demonstrated that methicillin resistance lowers Agr activity (41, 42), and as such, we tested whether the effect on daptomycin susceptibility reported here was also due to the expression of *mecA* suppressing Agr activity and the resulting production of PSMs. First, we measured the hemolytic ability of the strains as a proxy for Agr activity. This demonstrated that, as expected, the MSSA strains were more hemolytic than the MRSA strains, indicating that they had higher levels of Agr activity (Fig. 6A). Additionally, this was dependent on *mecA,* as the *mecA*::Tn mutant was more hemolytic than the WT strain (Fig. 6B), and complementation of the MSSA strains with p*mecA* led to a reduction in hemolysis compared with the empty plasmid controls (Fig. 6C).

Following on from this, we visualized the amount of PSMs released into the supernatant using SDS-PAGE, where PSMs (molecular weight 2–5 kDa) migrate as a band ahead of the bromophenol blue dye front (43). As expected, this agreed with the hemolysis data, with the MSSA strains producing more PSMs than MRSA strains (Fig. 6D), the *mecA*::Tn mutant producing more PSMs than the WT strain (Fig. 6E) and complementation of the MSSA strains with p*mecA* leading to a decrease in PSM production compared with the empty plasmid controls (Fig. 6F).

As an additional confirmation that the reduced daptomycin inactivation and subsequent decreased daptomycin tolerance were due to the release of PSMs, the JE2 WT strain (which produces relatively lower amounts of PSMs) was exposed to daptomycin in spent culture supernatant from either the WT strain or the *mecA*::Tn mutant strain (which produces relatively higher levels of PSMs). The WT strain was less tolerant of daptomycin when antibiotic exposure occurred in the presence of supernatant from the *mecA*::Tn mutant compared to supernatant from the WT strain, with over a nine-fold difference in survival observed at 6 h (Fig. 6G). Finally, we determined the effect of purified PSMs on the daptomycin tolerance of the WT strain and the *mecA*::Tn mutant (Fig. 6H through I). Strains were washed to remove any PSMs and other secreted factors that had been produced during the overnight growth before being exposed to daptomycin in fresh media supplemented, or not, with 10 µM purified PSMs. Due to the removal of bacterially-produced PSMs, both the WT strain and *mecA*::Tn mutant showed high levels of daptomycin tolerance (Fig. 6H through I). Addition of either PSMα2 or PSMα3, but not PSMα1, significantly reduced the daptomycin survival of both the WT and *mecA*::Tn mutant strains (Fig. 6H through I), demonstrating their ability to compromise bacterial survival upon exposure to daptomycin.

Having established the mechanism underlying the effect of *mecA* on daptomycin tolerance, our final step was to understand why the ASASM 330 strain did not behave as the other MSSA strains tested but instead showed rapid daptomycin inactivation and high survival (Fig. 3E and 5G). To investigate this, we examined the whole genome sequences of these strains, which had been generated previously (33). This revealed that ASASM 330 is the only strain in the panel of 165 CC30 isolates to have a nonsense mutation (C583T) in *agrC*, the gene encoding the sensor kinase of the Agr quorum sensing system and which controls PSM production. This strain is therefore an *agr* mutant and indeed phenocopies the *agrA* mutant examined in Fig. S3, explaining its high level of tolerance and low level of PSM production and daptomycin inactivation in the absence of *mecA*.

Taken together, the expression of *mecA* reduces Agr activity and the associated PSM production, enabling daptomycin to be inactivated by the released phospholipids and promoting bacterial tolerance of the antibiotic.

## DISCUSSION

*S. aureus* is a leading cause of bacteremia, and when the infection is caused by an MRSA strain, daptomycin is one of the few recommended treatment options. Unfortunately, daptomycin treatment is not always effective, failing in 20%–30% of cases and resulting in high mortality rates (17, 44). Here, we aimed to understand the molecular mechanisms underlying daptomycin tolerance as these may help us to understand why this treatment failure occurs, something that is crucial to developing improved approaches to manage staphylococcal infections. By testing the survival of 300 clinical bacteremia isolates on exposure to daptomycin, we identified *mecA* as a crucial determinant of daptomycin tolerance.

The *mecA* gene encodes the low affinity PBP, PBP2a, and confers resistance to beta-lactam antibiotics. It is encoded within the SCC*mec* element, a region of variable size, which can be excised and integrated into genomes using the CcrAB recombinases (45). The size of the SCC*mec* element varies depending on the clonal complex, with CC22 strains having a small type IV SCC*mec* element and CC30 having a large type II SCC*mec* element (46). Larger SCC*mec* elements contain integrated mobile genetic elements, such as plasmids or transposons, which can confer resistance to other antibiotics, including erythromycin, kanamycin, and tetracycline, and heavy metals, such as mercury and cadmium (46). However, to the best of our knowledge, this is the first time it has been linked to daptomycin tolerance.

It has previously been suggested that the presence of a large SCC*mec* element, such as that found in CC30, affects the fitness of the bacteria and compromises their ability to secrete toxins (41, 47). In addition, it has been shown that strains from CC30 are less toxic than those from CC22 (33), possibly explaining our observation that the CC22 strains were less tolerant of daptomycin than the CC30 strains and highlighting a trade-off between antibiotic susceptibility and toxicity. The trade-off between toxicity and beta-lactam resistance has been described previously; however, this work extends this to daptomycin as well. Additionally, the increased daptomycin tolerance of strains from CC30 compared with those from CC22 may suggest that infections caused by CC30 strains would be harder to eradicate with this antibiotic than those caused by CC22 strains. This emphasizes the importance of taking into account the genetic background of the strain when selecting an appropriate antibiotic treatment.

Daptomycin dosage is also known to affect treatment success rates, with higher doses (>6 mg/kg associated with better treatment outcomes than lower doses (<6 mg/kg) (16, 48). These higher doses can lead to trough plasma concentrations of approximately 30 µg mL^−1^ and peak concentrations of over 100 µg mL^-1^ (49, 50), considerably higher than the concentration of 10 µg mL^−1^ used in our study. However, daptomycin exhibits high levels of serum protein binding (over 90%) (50), and its activity is also affected by tissue penetration. For example, daptomycin is used to treat bone and joint infections and shows approximately 10% penetration into bone, resulting in bone concentrations of approximately 10 µg mL^-1^ (51), in line with the concentration used in this study. However, as we only tested a daptomycin concentration of 10 µg mL^−1^, it is possible that higher concentrations of daptomycin may be able to overcome the tolerance observed here.

The reason why the presence of a large SCC*mec* element suppresses toxin production is not known, although it has been suggested that PBP2a may cause modifications to the cell wall, which interfere with activation of the Agr quorum sensing system (41). To turn on Agr signaling, the autoinducing peptide (AIP) must bind to the membrane-bound AgrC sensor kinase (52). It is plausible that alterations in the cell wall could affect this. In support of this, removal of the cell wall with lysostaphin has been demonstrated to increase Agr activity in MRSA strains (41). We found that expression of *mecA* did not cause any differences in cell wall thickness, which could explain the altered Agr activity. However, it is possible that there are other changes within the peptidoglycan, such as altered crosslinking, or differences in the WTA component of the wall. Indeed, we observed that the surface of the MRSA strains was more positively charged than MSSA strains, and it is possible this was dependent on *mecA,* as there was a trend that the WT strain was more positively charged than the *mecA* mutant (although this did not reach statistical significance). This altered charge could affect the ability of AIP to traverse the wall and/or bind to AgrC.

Our results highlight that daptomycin may be more effective in treating MSSA than MRSA infections. MSSA infections are typically treated with anti-staphylococcal beta-lactams, such as nafcillin, oxacillin, and cefazolin; however, there are cases, for example, outpatient parenteral antibiotic therapy settings, where use of daptomycin may be preferable due to its once-daily administration. While daptomycin has been shown to be effective in treating MSSA infections, there has not been a clinical trial directly comparing treatment outcomes of patients infected with MRSA vs MSSA treated with daptomycin alone.

Daptomycin is often used in combination with a beta-lactam as they have been observed to be synergistic (53–55). Beta-lactams can increase the toxicity of *S. aureus* (56), and so, it is possible that this increased toxicity could enhance the activity of daptomycin, contributing to their synergistic relationship. By contrast, some antibiotics, including protein synthesis inhibitors such as clindamycin, are known to decrease bacterial toxin production (56). These could therefore promote the inactivation of daptomycin by phospholipids and so may be detrimental to treatment efficacy. The SNAP trial is currently testing whether, among other treatment options, the addition of clindamycin to a daptomycin/cefazolin combination improves patient outcomes in MRSA bacteremia compared with daptomycin and cefazolin (57).

In summary, in this study, we show that PBP2a is a crucial determinant of daptomycin tolerance. Strains expressing *mecA* produce lower amounts of PSMs, promoting daptomycin inactivation by phospholipids. Therefore, inhibition of PBP2a may be a viable strategy to enhance daptomycin efficacy for MRSA infection, improving patient outcome.

## MATERIALS AND METHODS

### Bacterial strains and growth conditions

The strains used in this study are shown in Table 1. The clinical isolates used are described in Recker et al. (33)*. S. aureus* was routinely grown on tryptic soy agar (TSA) plates at 37°C or in tryptic soy broth (TSB) at 37°C with shaking (180 rpm). Where appropriate, media were supplemented with 10 µg mL^−1^ erythromycin, 5 µg mL^−1^ chloramphenicol, or 10 µg mL^−1^ tetracycline. Strains carrying p*itet* or p*itet-mecA* were incubated with 200 ng mL^−1^ anhydrotetracycline.

**TABLE 1:** Bacterial strains used in this study

*S. aureus* strain	Relevant characteristics	Reference/ source
USA300 JE2 WT	Community-acquired MRSA strain of the USA300 lineage isolated from a skin and soft tissue infection at the Los Angeles County (LAC) jail, cured of plasmids	(37)
USA300 JE2 *mecA*::Tn	JE2 defective for PBP2a. Erm^R^	(37)
USA300 JE2 *mecA*::Tn p*itet*	JE2 defective for PBP2a carrying the empty p*itet* vector. Erm^R^/Cm^R^	This study
USA300 JE2 *mecA*::Tn p*mecA*	JE2 defective for PBP2a complemented with *mecA* in the p*itet* vector. Erm^R^/Cm^R^	This study
USA300 JE2 *agrA*::Tn	JE2 defective for AgrA. Erm^R^	(37)
CC30 EOE 29	Clinical MRSA bacteremia isolate from CC30	(33)
CC30 EOE 45	Clinical MRSA bacteremia isolate from CC30	(33)
CC30 EOE 120	Clinical MRSA bacteremia isolate from CC30	(33)
CC30 EOE 122	Clinical MRSA bacteremia isolate from CC30	(33)
CC30 EOE 205	Clinical MRSA bacteremia isolate from CC30	(33)
CC30 EOE 29 ΔSCC*mec*	EOE 29 with the SCC*mec* element deleted	This study
CC30 EOE 45 ΔSCC*mec*	EOE 45 with the SCC*mec* element deleted	This study
CC30 EOE 120 ΔSCC*mec*	EOE 120 with the SCC*mec* element deleted	This study
CC30 EOE 122 ΔSCC*mec*	EOE 122 with the SCC*mec* element deleted	This study
CC30 EOE 205 ΔSCC*mec*	EOE 205 with the SCC*mec* element deleted	This study
CC30 ASASM 43	Clinical MSSA bacteremia isolate from CC30	(33)
CC30 ASASM 47	Clinical MSSA bacteremia isolate from CC30	(33)
CC30 ASASM 78	Clinical MSSA bacteremia isolate from CC30	(33)
CC30 ASASM 99	Clinical MSSA bacteremia isolate from CC30	(33)
CC30 ASASM 330	Clinical MSSA bacteremia isolate from CC30	(33)
CC30 ASASM 43 p*itet*	Clinical MSSA bacteremia isolate from CC30 carrying the empty p*itet* vector. Cm^R^	This study
CC30 ASASM 47 p*itet*	Clinical MSSA bacteremia isolate from CC30 carrying the empty p*itet* vector. Cm^R^	This study
CC30 ASASM 78 p*itet*	Clinical MSSA bacteremia isolate from CC30 carrying the empty p*itet* vector. Cm^R^	This study
CC30 ASASM 99 p*itet*	Clinical MSSA bacteremia isolate from CC30 carrying the empty p*itet* vector. Cm^R^	This study
CC30 ASASM 330 p*itet*	Clinical MSSA bacteremia isolate from CC30 carrying the empty p*itet* vector. Cm^R^	This study
CC30 ASASM 43 p*mecA*	Clinical MSSA bacteremia isolate from CC30 carrying p*itet-mecA*. Cm^R^	This study
CC30 ASASM 47 p*mecA*	Clinical MSSA bacteremia isolate from CC30 carrying p*itet-mecA*. Cm^R^	This study
CC30 ASASM 78 p*mecA*	Clinical MSSA bacteremia isolate from CC30 carrying p*itet-mecA*. Cm^R^	This study
CC30 ASASM 99 p*mecA*	Clinical MSSA bacteremia isolate from CC30 carrying p*itet-mecA*. Cm^R^	This study
CC30 ASASM 330 p*mecA*	Clinical MSSA bacteremia isolate from CC30 carrying p*itet-mecA*. Cm^R^	This study
ATCC 29213 p*itet*	ATCC 29213 carrying the empty p*itet* vector. Cm^R^	This study
ATCC 29213 p*mecA*	ATCC 29213 carrying p*itet-mecA*. Cm^R^	This study

### Measurements of daptomycin tolerance

To measure the tolerance of the clinical isolates to daptomycin, strains were grown overnight in 1 mL TSB in bijou tubes at 37°C with shaking (180 rpm) before being diluted to 10^8^ CFU mL^−1^ in 1 mL TSB supplemented with 1.25 mM CaCl_2_ and 10 µg mL^−1^ daptomycin. Strains were incubated for 2 h at 37°C with shaking (180 rpm) before survival was determined by 10-fold serial dilutions in PBS and plating onto TSA.

In other cases, strains were grown overnight in 3 mL TSB in universal tubes before being diluted to 10^8^ CFU mL^−1^ in 3 mL TSB supplemented with 1.25 mM CaCl_2_ and 10 µg mL^−1^ daptomycin. Strains were incubated for 6 h at 37°C with shaking (180 rpm) before survival was determined by 10-fold serial dilutions in PBS and plating onto TSA. Where appropriate, spent culture supernatant was generated by growing *S. aureus* overnight in TSB and removing bacteria by centrifugation and filtration through a 0.2-µm filter. This spent culture supernatant was then supplemented with 1.25 mM CaCl_2_ and 10 µg mL^−1^ daptomycin before bacteria were added at 10^8^ CFU mL^−1^. When appropriate, overnights were washed twice in PBS before being diluted to 10^8^ CFU mL^−1^ in 3 mL TSB supplemented with 1.25 mM CaCl_2_, 10 µg mL^−1^ daptomycin and 10 µM purified PSMα1, PSMα2, or PSMα3.

### Determination of minimum inhibitory concentrations

MICs were determined using a broth microdilution protocol. Twofold serial dilutions of antibiotic were prepared in 200 µL TSB in a 96-well plate, then inoculated with 5 × 10^5^ CFU mL^−1^ and incubated statically at 37°C for 17 h. The MIC was determined as the minimum concentration with no visible growth. In the case of daptomycin, TSB was supplemented with 1.25 mM CaCl_2_. For determining the daptomycin MIC of the panel of clinical isolates, an agar macrodilution protocol was used. Twofold dilutions of daptomycin were prepared in TSA supplemented with 1.25 mM CaCl_2_ in square Petri dishes. Then, 10^4^ CFU per strain were spotted onto agar and incubated statically at 37°C for 17 h. The MIC was determined as the minimum concentration with no visible growth.

### Construction of p*mecA* and complementation of strains

The *mecA* gene was amplified from JE2 WT DNA using the primers avrII_mecA_Fw (5′- AGTCACCTAGGATGAAAAAGATAAAAATTGTTCCAC-3′) and pmeI_mecA_Rev (5′-AGTCAGTTTAAACCACTGTTTTGTTATTCATCTATATCG-3′). It was then digested with avrII and pmeI before being ligated into p*itet*, which had been similarly digested using T4 ligase. The plasmid was then transformed into *E. coli* DC10B, before being electroporated into *S. aureus* RN4220 and finally transduced into either the JE2 *mecA*::Tn mutant, the CC30 clinical strains, or ATCC 29213 using φ11.

### Deletion of the SCC*mec* element from clinical strains

The SCC*mec* element was deleted from the clinical strains as described previously (58). Briefly, the pSR2 plasmid, encoding the CcrAB recombinases, was transduced into the clinical strains at 30°C with tetracycline as the selection marker. The strain was sub-cultured in drug-free TSB for 24 h at 37°C and loss of the SCC*mec* element was confirmed by cloxacillin MIC.

### Determination of surface charge using FITC-PLL

Surface charge was measured as described previously (28). Briefly, 200-µL aliquots of overnight cultures were washed and resuspended in 200 µL PBS supplemented with 80 µg mL^−1^ FITC-PLL. Samples were incubated at room temperature in the dark for 10 min before being washed three times in PBS, resuspended in 200 µL PBS, and the fluorescence determined using a TECAN Infinite 200 PRO plate reader with an excitation of 485 nm and an emission of 525 nm.

### Quantification of cell-wall thickness by incorporation of HADA

Overnight cultures of *S. aureus* were generated in TSB supplemented with 25 µM HADA in the dark. Cultures were then washed three times in PBS, and the fluorescence was determined using a TECAN Infinite 200 PRO plate reader with an excitation of 405 nm and an emission of 450 nm.

### Measurement of membrane fluidity using laurdan

Overnight cultures of *S. aureus* were washed and diluted 10-fold in PBS. Aliquots (200 µL) were incubated in PBS supplemented with 100 µM laurdan for 5 min at room temperature in the dark. Samples were washed four times in PBS before membrane fluidity was determined by measuring fluorescence (excitation 330 nm; emission 460 and 500 nm) using a TECAN Infinite 200 PRO plate reader. The generalized polarization (GP) value was calculated using the formula: GP = (I_460_ − I_500_)/(I_460_+ I_500_), where I_460_ and I_500_ are the emission intensities, at 460 and 500 nm, respectively.

### Extraction and quantification of staphyloxanthin

Aliquots (1 mL) of overnight cultures of *S. aureus* were centrifuged and resuspended in 150 µL methanol. Samples were incubated for 30 min at 42°C before centrifugation, and the absorbance of the supernatant was determined at 462 nm.

### Extraction and quantification of WTA

Aliquots (1 mL) of overnight cultures of *S. aureus* were washed with 1 mL 50 mM MES (pH 6.5) (Buffer 1) and resuspended in 10 mL 50 mM MES (pH 6.5) supplemented with 4% SDS (Buffer 2). Samples were boiled for 1 h and centrifuged before being washed twice in Buffer 2, once in 50 mM MES (pH 6.5) supplemented with 2% NaCl (Buffer 3), and once in Buffer 1. The pellet was resuspended in 1 mL 20  mM Tris-HCl pH 8, 0.5% SDS, and digested with 20 µg proteinase K for 4 h at 50°C. The pellet was washed once with Buffer 3 and three times with water, then resuspended in 125 µL 0.1 M NaOH and incubated for 16 h at room temperature. After centrifugation, 100 µL supernatant was neutralized with 25  µl 1 M Tris-HCl (pH 7.8) and analyzed by PAGE. 10  µl aliquots of WTA samples were separated on a 20% native polyacrylamide gel by electrophoresis using 0.1 M Tris, 0.1 M Tricine, pH 8.2 running buffer. Gels were then stained with alcian blue (1  mg/mL in 3% acetic acid), destained with water, and visualized using a light box. Band intensity was quantified using ImageJ.

### Determination of antibacterial activity of daptomycin

Strains were grown overnight in 3 mL TSB in universal tubes before being diluted to 10^8^ CFU mL^−1^ in 3 mL TSB supplemented with 1.25 mM CaCl_2_ and 10 µg mL^−1^ daptomycin. At each time point, aliquots were taken and centrifuged at 13,000 x *g* for 1 min to remove the bacteria. Supernatants were filtered through 0.2 µm filters. MHA plates supplemented with 1.25 mM CaCl_2_ were overlaid with 100 µL of the JE2 WT strain at 10^7^ CFU mL^−1^, spread evenly over the surface of the agar with a spreader, then wells made in the agar using the wide end of a P1000 pipette tip and 100 µL supernatant added to the wells. All repeats of each assay were performed using one batch of MHA plates and each plate contained 20 mL agar to ensure uniform thickness. Plates were incubated for 16 h at 37°C before the diameters of the zones of clearance were measured. Four perpendicular measurements were taken per zone (from the edge of the well to the edge of the zone of clearance) and averaged. TSB supplemented with 1.25 mM CaCl_2_ and 10 µg mL^−1^ daptomycin with no bacteria added acted as a positive control, and values are presented as a percentage of the positive control or as zone size in millimeters.

### Determination of hemolysis

Aliquots (100µL) of supernatants from overnight cultures of *S. aureus* were serially diluted in twofold steps in TSB before being mixed with 100µL 2% defibrinated sheep blood in PBS. Samples were incubated statically for 1 h at 37°C before being centrifuged to remove unlysed erythrocytes. Supernatants (100µL) were moved to a fresh 96-well plate and absorbance determined at 450 nm. Erythrocytes incubated with TSB or TSB supplemented with 0.1% Triton-X100 were negative and positive controls, and values are presented as a percentage of the positive control.

### Measurements of PSM production

Equal volumes of supernatants from overnight cultures of *S. aureus* (grown to OD ~8, equivalent to ~1 × 10^9^ CFU mL^−1^) were analyzed by SDS-PAGE using 15% polyacrylamide gels in sample buffer containing bromophenol blue. The gels were stained using Coomassie blue, and the PSMs can be visualized as a band that runs ahead of the dye front. Relative band intensity was quantified using ImageJ.

### Statistical analyses

CFU data were log_10_ transformed and presented as the mean ± standard deviation. All experiments consisted of three or more independent replicates and were analyzed by *t*-test, one-way ANOVA or two-way ANOVA with appropriate *post-hoc* multiple comparison test using GraphPad Prism (V10.0), as described in the figure legends.

## SUPPLEMENTAL MATERIAL

Supplemental tables and figures mbio.02250-25-s0001.docx Tables S1 and S2 and Figures S1-S3.

ASM does not own the copyrights to Supplemental Material that may be linked to, or accessed through, an article. The authors have granted ASM a non-exclusive, world-wide license to publish the Supplemental Material files. Please contact the corresponding author directly for reuse.
