# Supplementary material for: Expression of mecA increases daptomycin tolerance in Staphylococcus aureus
Source: mBio. 2025 Sep 22;16(11):e02250-25. doi: 10.1128/mbio.02250-25 (PMC12607837; doi:10.1128/mbio.02250-25)
Supplement: Supplemental tables and figures — Tables S1 and S2 and Figures S1-S3. [file mbio.02250-25-s0001.docx]

**Expression of *mecA* increases daptomycin tolerance in *Staphylococcus aureus***

Elizabeth V. K. Ledger^1,2^, Mario Recker^3,4^ and Ruth C. Massey^1,2,5,*^

^1^ School of Microbiology, University College Cork, Cork, Ireland.

^2^ APC Microbiome Ireland, University College Cork, Cork, Ireland.

^3^ Centre for Ecology and Conservation, University of Exeter, Penryn Campus, Penryn, UK.

^4^ Institute for Tropical Medicine, University of Tübingen, Tübingen, Germany.

^5^ School of Cellular and Molecular Medicine, University of Bristol, Bristol, UK.

* For correspondence email: r.massey@ucc.ie

This file includes:

Supplementary tables 1-2

Supplementary figures 1-3

Table S1. Daptomycin MICs of clinical isolates used in this study

|  | CC30 | | | | | | CC22 | | | | | | All strains | |
| --- | --- | --- | --- | --- | --- | --- | --- | --- | --- | --- | --- | --- | --- | --- |
|  | MRSA | | MSSA | | Total | | MRSA | | MSSA | | Total | |  |  |
| Daptomycin MIC | N | % | N | % | N | % | N | % | N | % | N | % | N | % |
| 0.5 | 82 | 93.2 | 74 | 96.1 | 156 | 94.5 | 108 | 99.1 | 23 | 88.5 | 131 | 97.0 | 287 | 95.7 |
| 1 | 6 | 6.8 | 3 | 3.9 | 9 | 5.5 | 0 | 0.0 | 3 | 11.5 | 3 | 2.2 | 12 | 4.0 |
| 2 | 0 | 0.0 | 0 | 0.0 | 0 | 0.0 | 1 | 0.9 | 0 | 0.0 | 1 | 0.7 | 1 | 0.3 |
| Total | 88 | 100 | 77 | 100 | 165 | 100 | 109 | 100 | 26 | 100 | 135 | 100 | 300 | 100 |

| Strain | | | MIC (µg ml^-1^) | | |
| --- | --- | --- | --- | --- | --- |
|  |  |  | Cloxacillin | Cefoxitin | Daptomycin |
| MRSA |  |  |  |  |  |
| CC8 | JE2 | WT | 0.125 | 16 | 0.5 |
| CC8 | JE2 | *mecA*::Tn | 0.125 | 2 | 0.5 |
| CC8 | JE2 | *mecA*::Tn p*itet* | 0.125 | 2 | 0.5 |
| CC8 | JE2 | *mecA*::Tn p*mecA* | 0.125 | 8 | 0.5 |
| CC30 | EOE29 |  | 128 | 128 | 0.5 |
| CC30 | EOE45 |  | 256 | 128 | 0.5 |
| CC30 | EOE120 |  | 64 | 128 | 0.5 |
| CC30 | EOE122 |  | 128 | 128 | 0.5 |
| CC30 | EOE205 |  | >256 | >256 | 0.5 |
| CC30 | EOE29 | ΔSCC*mec* | 0.125 | 2 | 0.5 |
| CC30 | EOE45 | ΔSCC*mec* | 0.125 | 2 | 0.5 |
| CC30 | EOE120 | ΔSCC*mec* | 0.125 | 2 | 0.5 |
| CC30 | EOE122 | ΔSCC*mec* | 0.125 | 2 | 0.5 |
| CC30 | EOE205 | ΔSCC*mec* | 0.125 | 2 | 0.5 |
| MSSA |  |  |  |  |  |
| CC30 | ASASM43 |  | 0.0625 | 2 | 0.5 |
| CC30 | ASASM47 |  | 0.125 | 2 | 0.5 |
| CC30 | ASASM78 |  | 0.125 | 2 | 0.5 |
| CC30 | ASASM99 |  | 0.125 | 2 | 0.5 |
| CC30 | ASASM330 |  | 0.125 | 2 | 0.5 |
| CC30 | ASASM43 | p*itet* | 0.125 | 2 | 0.5 |
| CC30 | ASASM47 | p*itet* | 0.125 | 2 | 0.5 |
| CC30 | ASASM78 | p*itet* | 0.125 | 2 | 0.5 |
| CC30 | ASASM99 | p*itet* | 0.125 | 2 | 0.5 |
| CC30 | ASASM330 | p*itet* | 0.125 | 2 | 0.5 |
| CC30 | ASASM43 | p*mecA* | 0.125 | 2 | 0.5 |
| CC30 | ASASM47 | p*mecA* | 0.125 | 2 | 0.5 |
| CC30 | ASASM78 | p*mecA* | 0.125 | 2 | 0.5 |
| CC30 | ASASM99 | p*mecA* | 0.125 | 2 | 0.5 |
| CC30 | ASASM330 | p*mecA* | 0.125 | 2 | 0.5 |

Table S2. MICs of strains used in this study


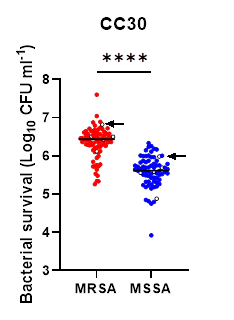


**Fig. S1. Daptomycin susceptibility of strains chosen for further characterisation**. Data from Fig. 1C with the strains chosen for further characterisation represented by white circles. The arrows point to EOE29 and ASASM330.

**Fig. S2. Expression of *mecA* in ATCC 29213 reduced daptomycin susceptibility.** Log10 CFU ml^-1^ of ATCC 29213 containing pEmpty (blue) or p*mecA* (red) during a 6 h exposure to 10 µg ml^-1^ daptomycin. Data represent the mean ± standard deviation of three independent biological repeats. Data were analysed by two-way ANOVA with Sidak’s *post-hoc* test. *, P < 0.05 (pEmpty vs p*mecA*).


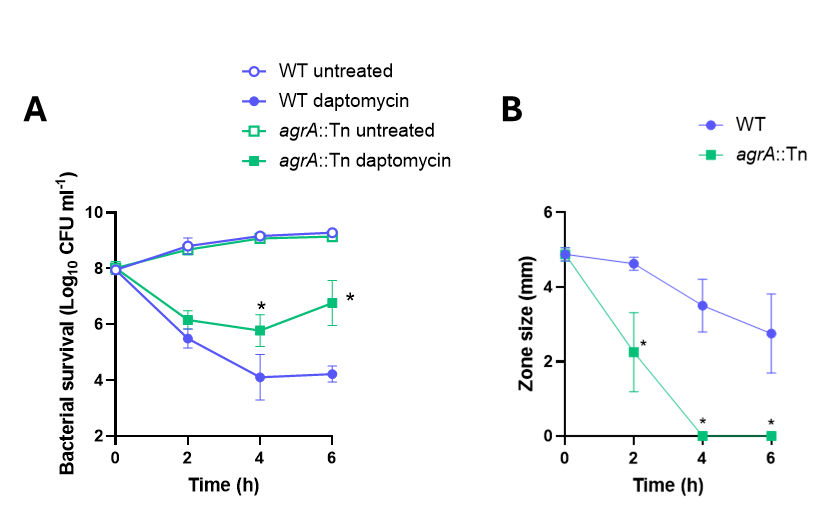


**Fig. S3. An *agrA*::Tn mutant shows reduced daptomycin susceptibility and increased inactivation of the antibiotic.** (A) Log10 CFU ml^-1^ of JE2 WT or the *agrA*::Tn mutant during a 6 h exposure to 0 (untreated) or 10 µg ml^-1^ daptomycin. (B) Daptomycin activity remaining in the supernatant after exposure of JE2 WT or the *agr*A::Tn mutant to 10 µg ml^-1^ daptomycin as measured by a zone of inhibition assay. Data represent the mean ± standard deviation of three independent biological repeats. Data were analysed by two-way ANOVA with Sidak’s *post-hoc* test. *, P < 0.05 (WT vs *agrA*::Tn).
